# High fat diet-induced non alcoholic fatty liver disease in rats is associated with hyperhomocysteinemia caused by down regulation of the transsulphuration pathway

**DOI:** 10.1186/1476-511X-10-60

**Published:** 2011-04-19

**Authors:** Elena Bravo, Simonetta Palleschi, Patricia Aspichueta, Xabier Buqué, Barbara Rossi, Ainara Cano, Mariarosaria Napolitano, Begoña Ochoa, Kathleen M Botham

**Affiliations:** 1 Department of Cellular Biology and Neuroscience, Istituto Superiore Sanità, Rome, Italy; 2 Department of Hematology, Oncology and Molecular Medicine, Istituto Superiore Sanità, Rome, Italy; 3 Department of Physiology, University of the Basque Country Faculty of Medicine and Dentistry, Sarriena s/n, 48940 Leioa, Spain. Rome, Italy; 4 Department of Veterinary Basic Sciences, the Royal Veterinary College, Royal College St., London NW1 0TU, UK

**Keywords:** Homocysteine, hyperhomocysteinemia, non alcoholic fatty liver disease, transsulphuration pathway

## Abstract

**Background:**

Hyperhomocysteinemia (HHcy) causes increased oxidative stress and is an independent risk factor for cardiovascular disease. Oxidative stress is now believed to be a major contributory factor in the development of non alcoholic fatty liver disease, the most common liver disorder worldwide. In this study, the changes which occur in homocysteine (Hcy) metabolism in high fat-diet induced non alcoholic fatty liver disease (NAFLD) in rats were investigated.

**Methods and results:**

After feeding rats a standard low fat diet (control) or a high fat diet (57% metabolisable energy as fat) for 18 weeks, the concentration of homocysteine in the plasma was significantly raised while that of cysteine was lowered in the high fat as compared to the control diet fed animals. The hepatic activities of cystathionine β-synthase (CBS) and cystathionine γ-lyase (CGS), the enzymes responsible for the breakdown of homocysteine to cysteine via the transsulphuration pathway in the liver, were also significantly reduced in the high fat-fed group.

**Conclusions:**

These results indicate that high fat diet-induced NAFLD in rats is associated with increased plasma Hcy levels caused by down-regulation of hepatic CBS and CGL activity. Thus, HHcy occurs at an early stage in high fat diet-induced NAFLD and is likely to contribute to the increased risk of cardiovascular disease associated with the condition.

## Background

Non alcoholic fatty liver disease (NAFLD) is the most common liver disorder in the world, and in obesity, type 2 diabetes and related metabolic diseases, its incidence reaches 70-90% [1]. The disease is characterised by the accumulation of triacylglycerol (TG) inside liver cells, and the condition can progress into more serious liver disease, such as non alcoholic steatohepatitis (NASH), liver fibrosis, cirrhosis, and more rarely, liver carcinoma [1]. Although it is known that progression of the disease is more likely to occur in patients with metabolic diseases [2], the factors involved are not well understood. However, oxidative stress coupled with insulin resistance is believed to play an important role [3].

Current evidence indicates that insulin resistance causes oxidative stress via increased oxidation in the liver, raised formation of reactive oxygen species, higher levels of hepatic lipid peroxidation, protein oxidation and pro-inflammatory cytokine production and decreased antioxidant capacity in the plasma [3-7]. Since the hepatic steatosis in NAFLD is believed to follow from the development of insulin resistance, oxidative stress is now considered to be one of the major causes of NAFLD [8,9].

Recent studies have suggested that NAFLD is associated with accelerated atherosclerosis [10], but the underlying mechanisms are not well understood. Moderately raised plasma levels of homocysteine (Hcy) have been found to be associated with atherosclerosis development, and hyperhomocysteinemia (HHcy) is considered to be an independent risk factor for cardiovascular disease [11,12]. Hcy is a thiol-containing amino acid involved in the metabolism of methionine in the liver. Methionine from dietary sources is converted to S-adenosyl methionine (SAM) by methionine adenosyl transferase (MAT). SAM is the methyl donor in most biological methylation reactions, and various methyltransferases (eg phosphatidyl-ethanolamine N-methyltransferase (PEMT) and glycine N-methyltransferase (GNMT)) are involved in its usage for the formation of phospholipids, myelin, and other macromolecules [12]. The product of the methyl transfer reactions is S-adenosyl homocysteine (SAH), which is then hydrolyzed to Hcy. Once formed, Hcy may be either remethylated to methionine (methionine cycle) or metabolised to cysteine (Cys) in a two step pathway catalysed by the enzymes cystathionine β-synthase (CBS) and cystathionine γ-lyase (CGL) (the transsulphuration pathway) [12,13]. HHcy is known to be associated with atherosclerosis and other pathologies, and oxidative stress, due to NADPH oxidase or NO synthase dependent generation of superoxide anion combined with a decrease in antioxidant enzyme activity, is thought to be a major factor in its effects [11,12]. In particular, when HHcy is due to disorders of the transsulphuration pathway, it associates with a reduced supply of Cys for the formation of the antioxidant glutathione (GSH) [13]. It has been reported that HHcy caused by CBS deficiency leads to disturbances in the regulation of lipid metabolism and fat accumulation in the liver [14,15]. Moreover, plasma Hcy is elevated in patients with NAFLD, and is a predictor of steatohepatitis [16].

Our previous work has shown that feeding rats a high fat diet (57% of energy from fat) induces insulin resistance, hypertriglyceridemia, hepatic steatosis and liver damage, which are characteristic of NAFLD and thus provides a suitable model for the early stages of the disease [17,18]. In the present study, we have used this model to test the hypothesis that high fat diet-induced NAFLD in rats modifies hepatic Hcy metabolism via modulation of the transsuphuration pathway.

## Methods

### Animals and diets

Male Wistar rats (200 g) (Harlan, S. Pietro al Natisone, Italy) were housed individually and allowed food and water *ad libitum*. All procedures conformed to the Guidelines of the European Community Council for animal care and use and to Decree 116/92, the Italian enforcement of the European Directive 86/609/EEC. The experimental protocol was approved by the Animal Care Ethics Committee of the Istituto Superiore di Sanità (ISS) and, according to the national law requirement the communication of the study has been sent by the ISS to the Animal Welfare Office ( Office VI) of the Italian Minister of Health (Communication Protocol Number 983/SSA/07). Rats were fed a standard low fat diet (4.3% fat, 10% of the metabolizable energy; control diet, 6 rats) or a diet containing 35% fat (31.6% saturated fat and 3.2% unsaturated fat, 57% of the metabolizable energy [18]; high fat diet, 4 rats) (Mucedola srl, Settimo Milanese, Italy) for 18 weeks. All rats were then sacrificed after fasting overnight. Blood samples were collected via heart puncture with the rats under terminal anesthesia (Acepromazine 2.5 mg/kg + Xylazine 1 - 5 mg/kg im) and centrifuged (3,500 rpm, 15 min, 6°C) to obtain the plasma. Livers were excised, washed with cold physiological saline (0.9%), samples (200 mg) were homogenised in methanol (5 ml) and the lipids extracted [19].

### Determination of CBS and CGL activities

CBS activity was assayed by a modification of the procedure of Mudd et al. [20]. Liver tissue (200 mg) was homogenized in ice-cold KH_2_PO_4 _(50 mM, pH 7.5), centrifuged (14,000 *g*, 10 min, 4°C) and the supernatant collected. L-Hcy (3 μmol) was added to the reaction mixture (100 mM Tris, pH 8.6; pyridoxal 5'-phosphate, 0.2 μmol; liver 14,000 g supernatant, 400-600 μg protein; 0.5 mg/ml BSA; L-[3-^14^C]serine (GE Healthcare, Amersham, UK), 0.06 μCi; serine, 2 μmol; L-cystathionine, 0.2 μmol) and incubated at 37°C for 90 min. The reaction was terminated with trifluoroacetic acid (10%, 200 μl). After centrifugation (10,000 *g*, 5 min), the [^14^C]cystathionine product was isolated using AG-50W-X8 resin (Bio-Rad) and eluted with 6 M NH_4_OH. Radioactivity was determined by scintillation counting. The assay was linear with time and protein concentration in the ranges used. 1 unit of CBS = that forming 1 nmol cystathionine per hour at 37°C. CGL activity was measured in cytosol as in [21]. Liver tissue (200 mg) was homogenized in ice-cold KH_2_PO_4 _(30 mM, pH 6.9), centrifuged (28,000 *g*,30 min, 4°C) and the supernatant collected. Cystathionine (2 μmol) was added to the reaction mixture (Tris-HCl buffer, pH 8.4, 50 μmol; pyridoxal 5'-phosphate, 0.125 μmol; liver 28,000 *g *supernatant, 400-600 μg protein) and incubated for 30 min at 37°C. After placing the tubes in ice to terminate the reaction, dithiothreitol (5 μmol/tube) was added. Cys was determined spectrophotometrically using acid ninhydrin reagent (1:1, v:v). The amount of Cys formed was linear with time and protein concentration in the ranges used. 1 unit of CGL = that forming 1 nmol of Cys per min at 37°C.

### Determination of mRNA abundance

mRNA abundance for mat type I, alpha (mat1a), pemt and gnmt in liver tissue was determined by qPCR. Total RNA was extracted using a GenElute Mammalian Total RNA kit (Sigma, Poole, Dorset, UK). First-strand cDNA synthesis was carried out using a Reverse Transcription System kit (Promega, Madison, WI, USA) and qPCR was performed in a DNA Engine Opticon 3 Light cycler (MJ Research, GMI Inc, Ramsay, MN, USA) using SYBR Green Jump Start Taq Ready Mix (Sigma, Gillingham, UK). Table 1 shows the forward and reverse oligonucleotide primers used. Conditions for PCR were; denaturation at 94°C for 2 mins; 37 cycles of 94°C for 15 secs; annealing at 56°C for 1 min; final extension of 72°C for 1 min; melting curve programme (60°C-95°C, heating rate 0.2°C/sec). The threshold cycle values were determined using Opticon Monitor 3 software quantified using the standard curve for each gene. Normalization was performed using the Visual Basic Application GeNorm [22].

**Table 1:** Primers used for qPCR

Gene name	Gene product	Gene Bank code/ID	Forward	Reverse
actb	β-Actin	NM_031144	gggaaatcgtgcgtgacatt	gcggcagtggccatctc

alb	Albumin	NM_134326	catcctgaaccgtctgtgtg	tttccaccaaggacccacta

b2m	β_2_-Microglobulin	NM_012512	atctgaggtgggtggaactg	tgaccgtgatctttctggtg

Mat1a	MAT-1A	BC089770	cacccaggctacctggtaaa	ccttcaaggctttcttgtgc

pemt	PEMT	NM_013003	ctcccaccttgctaccacat	agctcccatttccttctggt

gnmt	GNMT	NM_017084	ggtgctcactctggtcacct	gcctttgacaagtgggtcat

### Analytical methods

The triacylglycerol (TG) and cholesterol content of plasma and liver lipid extracts was assayed using enzyme-based kits (BPC BioSed S.r.l., Rome, Italy). Plasma insulin was measured by ELISA (Mercodia, Uppsala, Sweden). Protein concentrations were determined using commercial bicinchoninic acid reagent (Pierce) (liver supernatants) or the method of Bradford [23]. Plasma Hcy and Cys concentrations were measured by HPLC with fluorometric detection [24].

Significance limits were calculated using Student's t test.

## Results and discussion

### Results

**Figure 1:**
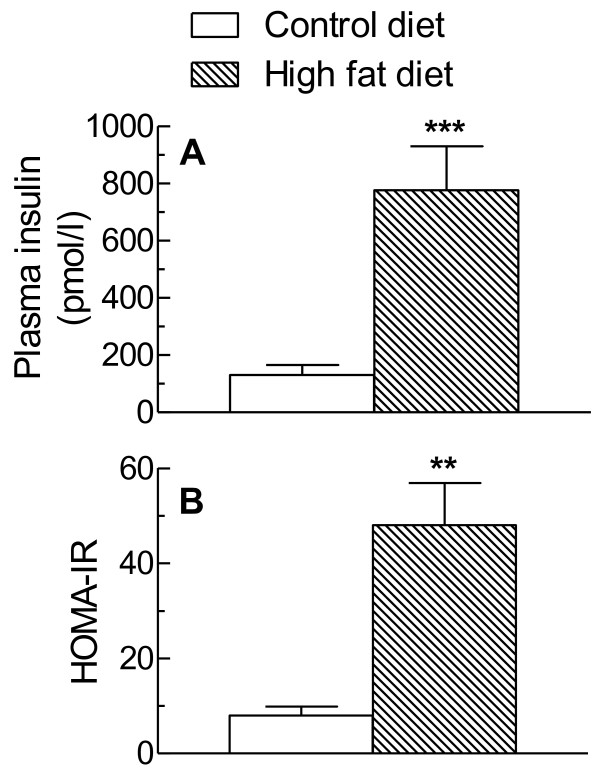
**Effect of high fat diet feeding on plasma insulin and HOMA-IR**. Rats were fed standard low fat diet (Control diet) or a high fat diet for 18 weeks. Blood samples were then collected and the concentration of insulin in plasma (A) were determined and the HOMA-IR (B) calculated. Data are the mean from 6 (Control diet) or 4 (High fat diet) animals and error bars show the SEM. **P < 0.01, ***P < 0.001 vs Control diet.

Liver TG concentrations were increased by about 2.6 fold in the rats fed the high fat diet as compared to the control diet and there was a more modest, but significant increase (+30%) in liver cholesterol levels (Table 2). In rats fed the high fat diet plasma insulin levels were about 6 fold higher than in animals given the control diet (Figure 1A) and the HOMA-IR showed a rise of a similar magnitude (Figure 1B). Plasma glucose levels, however, were not significantly changed (control diet, 8.75 ± 0.70 mmol/l; high fat diet, 8.45 ± 0.27 mmol/l).

**Table 2:** Effect of high fat diet feeding on plasma TG and cholesterol levels

Diet	TG (mg/g liver)	Cholesterol (mg/g liver)
Control diet	19.7 ± 4.8	31.8 ± 1.0

High fat diet	51.7 ± 5.6**	40.8 ± 2.6**

Rats were fed standard low fat diet (Control diet) or a high fat diet for 18 weeks. Blood samples were then collected and the concentration TG and cholesterol in plasma was measured. Data are the mean from 6 (Control diet) or 4 (High fat diet) animals. **P < 0.01 vs Control diet.

**Figure 2:**
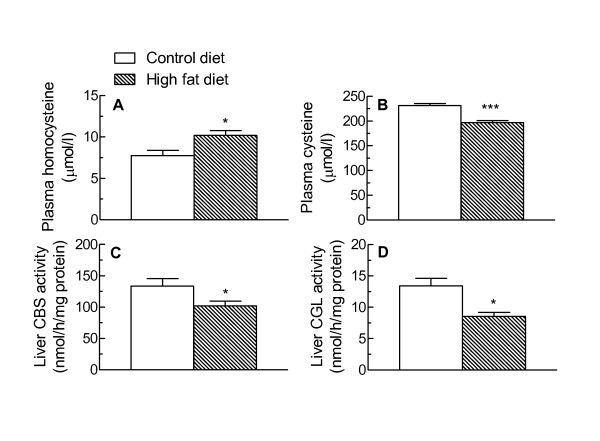
**Effect of high fat diet feeding on plasma homocysteine and liver CBS and CGL activities**. Rats were fed a standard low fat (Control diet) or a high fat diet for 18 weeks and blood samples and livers were then collected. A. Plasma Hcy; B. Plasma Cys; C. Liver CBS activity; D. Liver CGL activity. Data are the mean from 6 (Control diet) or 4 (High fat diet) animals. Error bars show the SEM. *P < 0.05, ***P < 0.001 vs Control diet.

Plasma Hcy and Cys concentrations in control diet- and high fat diet -fed rats are shown in Figure 2. Hcy concentrations in the plasma of the rats fed the high fat diet were significantly higher than those in control animals (+31%, P < 0.05) (Figure 2A), and this increase was accompanied by a decrease in plasma Cys levels (-15%, P < 0.001) (Figure 2B). In addition, the activities of both CBS and CGL in the liver were significantly reduced by high fat feeding (Figure 2). CBS activity was decreased by about 15% (P < 0.05) (Figure 2C), while that of CGL was lowered by approximately 23% (P < 0.05) (Figure 2D).

Determination of the relative abundance of mRNA transcripts for MAT1A, PEMT and GNMT showed no significant changes between the control and high fat-fed groups (Table 3).

**Table 3:** Effect of high fat diet feeding on expression of mRNA for methyltransferases

Gene	Control diet		High fat diet	
	**mRNA abundance/n factor/10,000**	**SEM**	**mRNA abundance/n factor/10,000**	**SEM**

mat1a	6.60	1.10	4.96	0.51

pemt	6.32	0.44	7.27	0.64

gnmt	6.26	1.01	6.81	0.70

Expression of mRNA for mat1a, pemt and gnmt. Rats were fed standard low fat diet (Control diet) or a high fat diet for 18 weeks. The livers were then excised, total mRNA was extracted and the relative abundance of transcripts for mat1a, pemt and gnmt was assessed by qPCR and expressed as mRNA abundance/normalisation factor (n factor)/10,000. Data are the mean from 7 (Control diet) or 10 (High fat diet) animals.

### Discussion

Oxidative stress is now believed to be an important factor in the development of NAFLD [3-5] acting as a cause as well as a consequence of hepatic steatosis [6,25]. Here, we investigated the changes which occur Hcy metabolism in a high fat-diet induced model of NAFLD in rats. The high fat diet used caused an increase in liver TG ( × 2.6) and cholesterol (+ 30%) and plasma insulin levels and the HOMA-IR were increased by 6 fold (Figure 1). These results are in agreement with our previous more extensive characterisation of the effects of this diet in inducing NAFLD [17], and indicate that the animals are a good model for the condition.

A number of studies have implicated HHcy in premature atherosclerosis development, and oxidative stress is thought to be a major factor in this effect [11,12]. NAFLD is associated with accelerated atherosclerosis [10], and oxidative stress is now believed to be an important factor in the development/progression of the condition [3-5]. Recent studies have indicated that increased oxidative stress is an important trigger for insulin resistance, which is believed to cause the disturbances in liver lipid metabolism that result in NAFLD. The resulting fat accumulation is then thought to promote further production of reactive oxygen species, thus oxidative stress can be considered to be both a cause and a consequence of hepatic steatosis [1,6,25]. In the liver, Hcy is produced from SAH formed during methyl transfer reactions involving SAM and methyltransferase enzymes such as GNMT and PEMT, and removed by conversion to Cys via the transsulphuration pathway which involves CBS and CGL [11-13]. Although it has been shown that the plasma Hcy levels are elevated in alcoholic fatty liver disease [26] and that HHcy is associated with the development of hepatic steatosis in mice deficient in CBS and in human subjects with a genetic defect in the enzyme [14-16,27], few studies have investigated Hcy metabolism in NAFLD. Gulsen et al. [16] have reported that plasma Hcy concentrations were significantly higher in NAFLD patients as compared to healthy subjects, and that they were a good predictor of progression to NASH. In contrast, however, another study found no significant difference in plasma Hcy levels in obese subjects with or without NAFLD [28].

The results of our experiments show that plasma Hcy is significantly increased in high fat diet-induced NAFLD in rats, and that this change is accompanied by a decrease in plasma Cys (Figure 2A,B), suggesting that the transsulphuration pathway is affected. Determination of the activities of the two enzymes of the pathway showed that both are significantly down-regulated in high fat diet-induced NAFLD (Figure 2C,D). We found no evidence, however, for changes in the expression of other key enzymes in the hepatic methionine cycle, including MAT1A, the gene which encodes the catalytic subunit of the isoenzymes MATI and MATIII which are expressed in adult liver [29], and the methyltransferases GNMT and PEMT (Table 3). Since these enzymes are methyltransferases while CBS and CGL are lyases, it is likely that they are regulated by different mechanisms. In addition, we have only measured mRNA expression for these enzymes, thus we cannot rule out the possibility that there may be post transcriptional changes which modulate their activity in response to high fat feeding. In contrast to our results, Kwon et al. [30] have reported that CBS activity was unchanged in rats in which NAFLD was induced by feeding a diet containing 71% of the energy as fat, while CGL activity was increased by about 35%. In this study, however, the control diet contained 35% of energy from fat, which is considerably higher than the 10% of energy from fat in our control diet. Thus, our findings indicate that high fat diet-induced NAFLD is associated with HHcy, and that this is caused by reduced conversion to Cys via the transsulphuration pathway, while the expression of methyltransferases involved in liver methionine metabolism is not changed. As it has been estimated that as much as 50% of the Cys required for GSH synthesis is formed from Hcy via this route, and the availability of Cys is a limiting factor for GSH production [12,31], in the long term this may lead to a decrease in body levels of this antioxidant.

## Conclusions

In conclusion, these results reported here show that high fat diet-induced NAFLD in rats causes HHcy and that this is due to down-regulation of hepatic CBS and CGL activity. Thus, HHcy is an early feature of high fat diet-induced NAFLD and is likely to contribute to the increased risk of cardiovascular disease associated with the condition.

## Abbreviations

CBS: cystathionine β-synthase; CGL: cystathionine γ-lyase; GNMT: glycine N-methyltransferase; GSH: glutathione; Hcy, homocysteine; HHcy: hyperhomocysteinemia; MAT: methionine adenosyl transferase; NAFLD: non alcoholic fatty liver disease; NASH: non alcoholic steatohepatitis; PEMT: phosphatidylethanolamine N-methyltransferase; SAM: S-adenosyl methionine.

## Competing interests

The authors declare that they have no competing interests.

## Authors' contributions

PA, XB, BR, AC, MN carried out experimental work; SP, EB, BO, KMB, designed and planned the study, and also drafted and critically revised the manuscript. All the authors contributed to the interpretation and discussion of results related to their part of the work and read and approved the final manuscript.
